# Tuberculosis in Cows

**Published:** 1900-03-24

**Authors:** 


### TUBERCULOSIS IN COWS.

In a discussion at the Edinburgh Medico-Chirurgical Society on the effect of heredity on disease, a somewhat interesting remark was made by Professor Stewart Stockman, who said that out of the 8,000 carcases of beasts slaughtered in the year at Edinburgh, 4 per cent. of the cows were tuberculous, while not 1 per cent. of the bullocks slaughtered there was affected with the disease. This he mentioned to show that if tuberculosis were to be regarded as hereditary, some explanation must be given of its being transmitted to the one sex more than to the other. The fact, however, is equally good as showing the importance of the different treatment dealt out to cows and bullocks respectively, especially in regard to prolonged confinement in ill-ventilated byres, in determining the greater proclivity of cows to tuberculosis.

